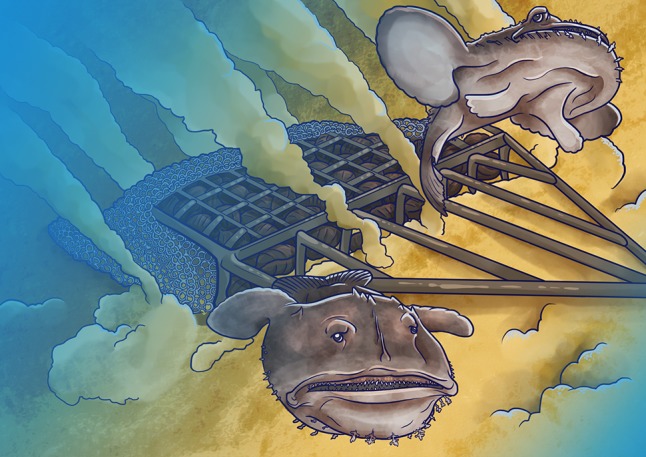# Caught out! Scientists discover a cryptic stress response in a bycatch species

**DOI:** 10.1093/conphys/coz008

**Published:** 2019-02-27

**Authors:** Essie M Rodgers

**Affiliations:** University of Antwerp, Systemic Physiological and Ecotoxicological Research (SPHERE), Campus Groenenborger, Groenenborgerlaan 171, Antwerp, Belgium

Every day, an overwhelming number of marine organisms are unintentionally hauled out of the oceans during fishing operations. These accidental captures, termed bycatch, impact a wide range of organisms including delicate corals, fish, turtles, dolphins, and even whales. Succumbing to capture can be stressful—organisms often face physical injury, air exposure, and drastic changes in temperature and pressure. In fact, new research has found that even fish that appear unharmed following accidental capture are highly stressed and unlikely to survive once released.

This finding was uncovered when Amelia Weissman and her colleagues (2018) set out to investigate the stress response of a commercially valuable fish, the monkfish (*Lophius americanus*), when accidentally captured while dredging for scallops. The gear used for scallop dredging consists of a metal frame, a collection net, and a toothed bar for dislodging scallops from the seabed. Boats tow this equipment across the seafloor to efficiently capture scallops, as well as any other organisms along the way.

The research team took to the ocean for several scallop dredge expeditions. They started by assessing the injuries the monkfish incurred during capture and paid particular attention to how long the fishing gear was towing through the water and how long the fish were exposed to air once captured. To their surprise, most of the fish were uninjured. However, as physiologists, the researchers knew they needed to delve deeper. They examined the fish’s reflex responses, and they also took blood samples to measure lactate and cortisol, both of which can indicate stress. A loss of critical reflex responses (jaw closure, eye fixation, spinal arching and body flexing) can indicate that a fish is about to die and would therefore not likely survive being released back into the ocean.

By delving deeper, Weissman and her team uncovered a cryptic stress response that wasn’t obvious from a simple visual assessment. Although captured fish showed minimal physical injuries, their biochemical stress markers (e.g., cortisol) soared. And, in some cases, levels were 100 times higher than in unstressed control fish! The team also discovered that the duration of the fishing gear towing events—especially those longer than 70 minutes—and how long the fish were exposed to air—especially if it was for longer than 20 minutes—also impacted cortisol levels.

These findings highlight just how stressful being hauled out of the ocean can be, even if a species is resilient to physical injuries. Shortening fishing gear towing durations is one strategy to reduce stress in bycatch species, but it can be difficult to track how long individual animals have been trapped in fishing gear. Alternatively, minimizing the time fish are exposed to air once they are captured can be easily achieved by immediately returning monkfish (and other animals) to the ocean or to a temporary recovery tank. Weissman’s study highlights the true importance of delving beyond outward appearances. Often, it’s what’s on the inside of an animal that tells us the most about its health.

Illustration by Erin Walsh; Email: ewalsh.sci@gmail.com

**Figure:**